# Methods of Secondary Smile Salvation After a Failed Primary Salvation Procedure for Facial Nerve Palsy: A Systematic Review

**DOI:** 10.1097/SAP.0000000000004776

**Published:** 2026-06-04

**Authors:** Hamish Thomson, Roya Fazliani, Embiye Adala, Christopher Jones

**Affiliations:** a School of Medicine, University of Liverpool, Liverpool; b The Mersey Regional Burns and Plastic Surgery Unit, Mersey and West Lancashire Teaching Hospitals NHS Trust, Knowsley, UK

**Keywords:** secondary smile salvation, revision, facial nerve palsy, free flap, smile correction

## Abstract

The smile is a fundamental facial expression that is important for emotional wellbeing. Long-term facial nerve palsy leads to an inability to smile, which can have profound effects on mental health. Although numerous techniques exist for facial reanimation, evidence on secondary interventions following a failed primary procedure remains scarce. This systematic review, conducted in accordance with PRISMA guidelines, aimed to identify techniques for secondary smile restoration. A systematic search of PubMed, Scopus, and MEDLINE identified 41 studies; after independent screening using PICOT-based inclusion and exclusion criteria, and a subsequent quality assessment, 4 studies (12 patients) were included. Eight distinct secondary techniques were reported, all of which were associated with improvement in smile-related outcomes and/or facial symmetry, alongside reduced House-Brackmann scores. However, these findings are limited by the small sample size, methodological shortcomings, and incomplete reporting, precluding an indication of the optimal technique. Future high-quality studies, particularly long-term randomised controlled trials with larger cohorts, and comprehensive reporting of patient characteristics and clinical outcomes, are required to establish the most effective method of secondary smile restoration.

The smile is a facial expression achieved by the coordinated stimulation of several facial muscles, neural circuits, sensory feedback loops, and emotional states. It is regarded as being in a state of happiness, euphoria and/or pleasure, and thereby, important for communicating. The facial nerve (CN. VII) stimulates the zygomaticus major/minor muscles to elevate the corners of the mouth, the orbicularis oculis to facilitate periocular creasing, and the levator anguli oris, risorius and buccinator to refine the smiles shape.^1,2^ Facial nerve palsy weakens or paralyzes the aforementioned muscles resulting in the inability to smile. In persistent cases of facial nerve palsy, quality of life can be significantly reduced through the consequential development of neuropsychiatric sequelae, including depression, anxiety, negative self-perception, and social withdrawal.^3,4^ In addition, evidence shows that the inability to smile itself may reduce the positive feedback effect that smiling provides, having a further detrimental impact on the patients mental health.^5^

Free functional muscle transfer, using the free gracilis muscle, is the standard reconstructive technique for long-standing facial nerve palsy where the native muscles of facial expression have atrophied. The flap is revascularised by microvascular anastomosis, and reinnervated either by the masseteric nerve, a cross-facial nerve graft (CFNG), or dual innervation. While these methods can restore the smile, it is not always successful. Flap failure may occur for many reasons, a nonexhaustive list includes concurrent radiotherapy, synkinesis, inadequate tension, or poor reinnervation, particularly in the event of long denervation time, or, donor nerves with low axon counts.^6,7^ Little evidence exists in the literature on methods of secondary salvation of the smile when the primary facial reanimation procedure is unsuccessful, identifying the need for a systematic review.

The primary objective of this review is to systematically review what is available in current literature on the current techniques of secondary smile augmentation, following a failed primary salvation procedure for facial nerve palsy. The results will guide decision-making for the management of a persistent inability to smile due to facial nerve palsy, following a failed primary reanimation attempt.

## METHODOLOGY

This is a systematic review adhering to PRISMA guidelines,^8^ and following the PICOT (population, intervention, comparison, outcome, time) framework,^9^ to evaluate the current evidence surrounding existing methods of secondary smile salvation, following a failed reanimation attempt for facial nerve palsy.

A systematic search was performed on 23rd September 2025 for all papers utilizing methods of secondary smile salvation. Articles were selected from the PubMed,^10^ Scopus^11^ and MEDLINE^12^ databases, with the MEDLINE search being performed using the OVID search engine.^13^ The authors conducted the search independently. Four groups of keywords were used: the first relating to facial nerve paralysis, the second to free flap reconstruction, the third relating to augmentation, and the fourth to the smile. Specific search terms and Boolean operators are presented in Tables 1–3. All study types were included in the search strategy. Following the search, all identified studies were uploaded to the latest version of the artificial intelligence systematic review software, Rayyan,^14^ and 2 authors (H.T. and R.F.) screened the abstracts, evaluating each papers’ suitability for inclusion in the review. In addition, each article’s bibliography was also screened to identify other relevant studies.

**TABLE 1:** PubMed Search Strategy

Number	Term	Results
**#1**	(“Facial Paralysis” [Mesh]) OR (“Facial Nerve Injuries” [Mesh]) OR (facial nerve palsy [tiab]) OR (facial paralysis [tiab]) OR (facial palsy [tiab])	21,535
**#2**	(“Free Tissue Flaps”[Mesh]) OR (free flap [tiab]) OR (free tissue transfer [tiab]) OR (free muscle flap [tiab]) OR (functional free flap [tiab]) OR (free functional muscle transfer [tiab]) OR (gracilis flap [tiab])	17,282
**#3**	(secondary [tiab]) OR (revision [tiab]) OR (augmentation [tiab]) OR (enhancement [tiab]) OR (secondary procedure [tiab]) OR (secondary reanimation [tiab]) OR (salvage [tiab])	1,554,128
**#4**	(smile [tiab]) OR (smile reanimation [tiab]) OR (smile symmetry [tiab]) OR (oral commissure excursion [tiab]) OR (oral commissure movement [tiab]) OR (oral commissure [tiab]) OR (spontaneous smile [tiab]) OR (functional outcome [tiab]) OR (esthetic outcome [tiab])	45,297
**#5**	#1 AND #2 AND #3 AND #4	15

Date–September 23, 2025.

**TABLE 2:** Scopus Search Strategy

Number	Term	Results
**#1**	“facial nerve palsy” OR “facial paralysis” OR “facial palsy”	23,025
**#2**	“free flap” OR “free tissue transfer” OR “free muscle flap” OR “functional free flap” OR “free functional muscle transfer” OR “gracilis flap”	21,818
**#3**	“secondary” OR “revision” OR “augmentation” OR “enhancement” OR “secondary procedure” OR “secondary reanimation” OR “salvage”	4,030,810
**#4**	“smile” OR “smile reanimation” OR “smile symmetry” OR “oral commissure excursion” OR “oral commissure movement” OR “spontaneous smile” OR “functional outcome” OR “esthetic outcome”	104,826
**#5**	#1 AND #2 AND #3 AND #4	15

Date–September 23, 2025.

**TABLE 3:** MEDLINE Search Strategy

Number	Term	Results
**#1**	(facial nerve palsy or facial paralysis or facial palsy).mp.	20,109
**#2**	(free flap or free tissue transfer or free muscle flap or functional free flap or free functional muscle transfer or gracilis flap).mp.	14, 911
**#3**	(secondary or revision or augmentation or enhancement or secondary procedure or secondary reanimation or salvage).mp.	1, 880, 894
**#4**	(smile or smile reanimation or smile symmetry or oral commissure excursion or oral commissure movement or spontaneous smile or functional outcome or esthetic outcome).mp.	44,909
**#5**	#1 AND #2 AND #3 AND #4	11

Date–September 23, 2025.

The study selection process was guided using the PICOT framework:^9^

- Population (P)–Patients undergoing secondary smile salvation, following failure of primary smile salvation for facial nerve palsy.
- Intervention (I)–Methods of secondary smile salvation.
- Comparison (C)–Primary smile salvation procedures.
- Outcome (O)–House-Brackmann score,^15^ complications, time to reinnervation, the ability to smile, and whether the restored smile was spontaneous or voluntary.
- Time (T)–Clinical outcomes assessed in both short-term (≤1 y) and long-term (≥1 y) follow-ups.

Eligible studies were limited to English only and were based on describing techniques of restoring the ability to smile, following a failed primary reanimation procedure for facial nerve palsy. Only papers published between 1 January 2005 and 23 September 2025 were included to ensure alignment with modern reconstruction techniques. Each study was required to clearly describe the technique used to restore the ability to smile and provide data on clinical outcomes, particularly in the context of smile restoration. Furthermore, correspondences, letters or discussions to editors, literature reviews, or abstract-only publications were also excluded.

The initial search yielded a total of 41 studies: 15 from PubMed, 15 from Scopus and 11 from MEDLINE. Following the removal of 21 duplicate papers, 20 studies were independently screened by 2 authors (H.T. and R.F.) against the PICOT-aligned inclusion and exclusion criteria; of these, 13 were excluded. Screening the bibliographies of all articles identified one additional study, which was not captured by the initial search for unclear reasons.^16^ This resulted in 8 studies undergoing quality assessment using the risk of bias in non-randomized studies of interventions, version 2 (ROBINS-I V2) tool.^17^ Four studies were excluded during this process.

**FIGURE 1:**
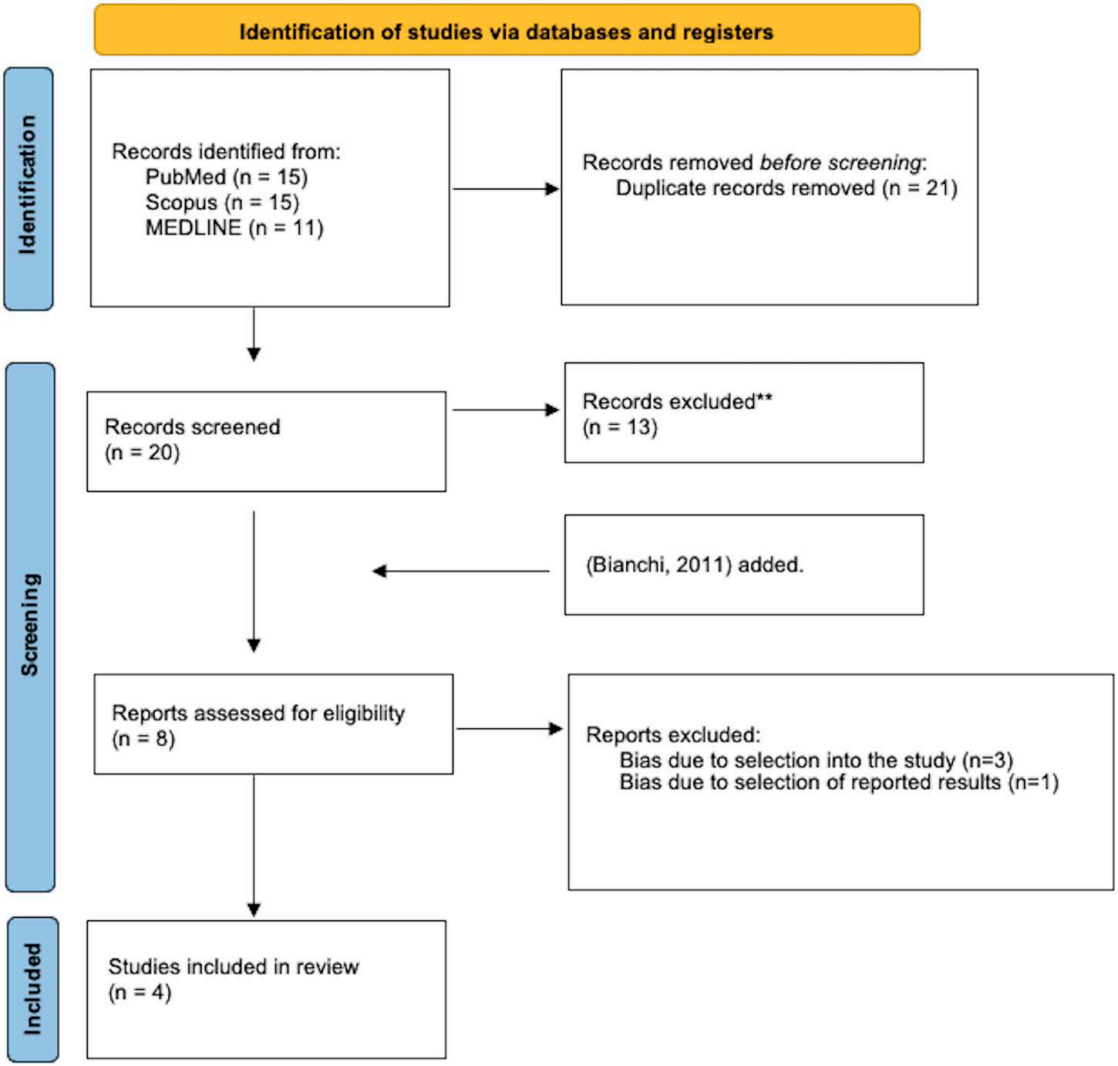
PRISMA flowchart.

Kollar et al^18^ scored a critical risk of bias in the selection of reported result domain, as they did not report outcomes in a patient-by-patient format, preventing the authors from extracting, and presenting data in the same way as other studies included in the review. Chi et al^19^ and Greene et al^20^ were excluded due to a critical risk of bias in the selection into the study domain, as their papers were centralized around patients’ aesthetic revisions following a successful primary reanimation procedure, such as thinning of the flap, thereby comprising a different patient cohort than that targeted by this review. Similarly, Wen et al^21^ was excluded as it focused on techniques for excising recurrent pleomorphic adenomas without compromising previous facial reanimation, rather than on management strategies for smile restoration following failed primary reanimation procedures. Following these exclusions, 4 studies were included in the final review. The study selection process is illustrated in Figure 1.

## RESULTS

The characteristics of each study, including location, design, patient demographics, and follow-up period, are summarized in Table 4. Owing to the scarcity of literature on secondary smile salvage, only 12 eligible patients were identified; therefore, the data are presented in a patient-by-patient format. The clinical characteristics of these patients, including age, sex, palsy laterality, aetiology and duration, suspected cause of primary salvation failure and time since primary salvation are outlined in Table 5. Details of both primary and secondary smile salvation procedures, as well as the associated clinical outcomes, are presented in Table 6.

**TABLE 4:** Study Characteristics

Study	Location	Single or Multi Center	Study Type	Sample, n	Age (y), Mean (Range)	Sex, n (%)	Follow-Up (m), Mean (Range) or (+/− SD)
Ibarra and Lasso^22^	Spain	Single	Retrospective	3	42 (27-56)	M – 2 (66.6) F – 1 (33.3)	NR
Mohanty et al^23^	USA	Single	Case series	4	59.3 (44-68)	M – 2 (50) F – 2 (50)	NR
Eisenhardt et al^24^	Germany	Single	Case series	3	39 (21-48)	F – 3 (100)	6±0
Bianchi et al^16^	Italy	Single	Case series	2	10 (8-12)	M – 2 (100)	10 (8-12)

NR indicates not reported.

**TABLE 5:** Patient Characteristics

Patient	Age	Sex	Laterality	Duration of Paralysis (y)	Aetiology for Primary Surgery	Previous Radiotherapy	Suspected cause of Primary Reanimation Failure	Time Since Primary Reanimation (y)
Patient 1	27	F	L	8	SCC	Yes	EBR	8
Patient 2	56	M	R	4	MEC	Yes	Cholesteatoma	4
Patient 3	43	M	L	7	MEC	Yes	EBR	3
Patient 4	61	F	L	4	BP	No	UC	NR
Patient 5	68	M	R	10	FNS	Yes	NC	NR
Patient 6	64	F	R	10	APC	No	UC	NR
Patient 7	44	M	R	0.75	ACC	Yes	CR	NR
Patient 8	21	F	NR	6	AN	No	NR	0.5
Patient 9	48	F	NR	1	AN	No	NR	0.6
Patient 10	48	F	NR	2	AN	No	NR	0.6
Patient 11	12	M	L	12	MS	No	UC	4
Patient 12	8	M	L	8	BP	No	Flap necrosis	NR

ACC indicates adenoid cystic carcinoma; AN, acoustic neuroma; APC, acinic parotid cancer; BP, Bell palsy; CR, cancer recurrence; EBR, external beam radiotherapy; FNS, facial nerve schwannoma; MEC, mucoepidermoid carcinoma; MS, Moebius syndrome; NC, neurotoxic chemotherapy; SCC, squamous cell carcinoma; UC, unclear.

**TABLE 6:** Primary and Secondary Smile Salvation Procedures

Patient	Primary Smile Salvation Procedure	House-Brackmann Score after Primary Smile Salvation Procedure	Secondary Smile Salvation Procedure	Secondary Recipient Vessels	Secondary Recipient Nerve; Anastomosis	House-Brackmann Score after Secondary Smile Salvation Procedure	Complications	Additional Procedures	Time to Reinnervation (d)	Ability to Smile	Smile Type
Patient 1	TRAM flap + FNCG using SN	VI	LDFF	A + V of previous TRAM anastomosis	PBDTN; E-T-E	III	NR	NR	NR	Yes	NR
Patient 2	GMFF + IIFNG	VI	SGMFF + MNC	A + V of previous GMFF anastomosis	MN; E-T-E	I	NR	NR	NR	Yes	NR
Patient 3	TPF	VI	GMFF + MNC	Contralateral FA + Contralateral FV	MN; E-T-E	III	NR	NR	NR	Yes	NR
Patient 4	GMFF + MNC	IV	Neurolysis	UC	UC	II	NR	SMFLS	8	Yes	NR
Patient 5	GMFF + MNC	VI	SGMFF + MNR	A + V of previous GMFF anastomosis	MN; E-T-E	III	NR	SMFLS	36	Yes	NR
Patient 6	GMFF + MNC	VI	SGMFF + MNR	A + V of previous GMFF anastomosis	MN; E-T-E	II	NR	SMFLS	64	Yes	Voluntary
Patient 7	GMFF + MNC + SMFLS	VI	SGMFF + MNR	VGs to contralateral neck vessels	MN; E-T-E	II	NR	NR	60	Yes	NR
Patient 8	GMFF + CFNG	VI	Coaptation of NP from CFNG to MN	FA + FV (UC)	MN; E-T-E	II	Hematoma	FT + CP	91.3	Yes	Voluntary
Patient 9	GMFF + CFNG	VI	Coaptation of NP from CFNG to MN	FA + FV (UC)	MN; E-T-E	NR	None	None	91.3	Yes	Voluntary
Patient 10	GMFF + CFNG	VI	Coaptation of NP from CFNG to MN	FA + FV (UC)	MN; E-T-E	NR	None	FT +SMFLS	91.3	Yes	Voluntary and spontaneous
Patient 11	GMFF + CFNG	VI	SGMFF	FA + FV	MN; E-T-E	III	None	None	91.3	Yes	Voluntary
Patient 12	LDFF + CFNG	VI	GMFF	TFA + TFV	MN; E-T-E	III	None	None	121.7	Yes	Voluntary

A indicates artery; AR, anastomosis revision; CFNG, cross-facial nerve graft; CP, canthoplasty; E-T-E, end to end; FA, facial artery; FNCG, facial nerve cable graft; FT, flap thinning; FV, facial vein; GMFF, gracilis muscle free flap; IIFNG, ipsilateral intrapetrous facial nerve graft; LDFF, latissimus dorsi free flap; MN, masseteric nerve; MNR, masseteric nerve recoaptation; NP, neural pedicle; NR, not reported; PBDTN, posterior branch deep temporal nerve; SGMFF, second gracilis muscle free flap; SMFLS, static midface fascia lata sling; SN, sural nerve; TFA, transverse facial artery; TFV, transverse facial vein; TPF, temporalis pedicled flap; TRAM, transverse rectus abdominis myocutaneous; UC, unchanged; V, vein; VG, venous graft; VP, vascular pedicle.

### Study Characteristics

Four studies were included in the final review, including 3 case series’^16,23,24^ and one retrospective cohort study.^22^ All papers were conducted in different countries, including the United States of America, Spain, Germany, and Italy. The mean sample size across the review was 3 (range 2 to 4). Male: female ratio across the review was 1:1. Eisenhardt et al^24^ reported a mean follow-up period of 6±0 months while Bianchi et al^16^ followed up patients for a mean of 10 months (range 8 to 12). Ibarra and Lasso^22^ and Mohanty et al^23^ failed to report the length of the follow-up period of their patients.

### Patient Characteristics

A total of 12 patients undergoing a secondary smile salvation procedure, following a failed primary salvation attempt for facial nerve palsy were identified. The mean age was 41.7 (range 8 to 68). Palsy laterality was on the left in 5 patients (41.7%), right in 3 patients (25%), and unreported in 3 patients (25%). The mean duration of paralysis before the secondary salvation attempt was 6 years (range 0.75 to 12). Aetiologies for facial nerve paralysis included acoustic neuroma (n=3), Bell palsy (n=2), mucoepidermoid carcinoma (n=2), squamous cell carcinoma (n=1), facial nerve schwannoma (n=1), adenoid cystic carcinoma (n=1), acinic parotid cancer (n=1), and Moebius syndrome (n=1). Five patients (41.7%) had received previous radiotherapy. The suspected causes of failure of the primary smile salvation included external beam radiotherapy (n=2), cholesteatoma (n=1), neurotoxic chemotherapy (n=1), cancer recurrence (n=1), flap necrosis (n=1), and was unreported in 6 patients (50%). The mean time between the primary and secondary salvation procedures was 3 years (range 0.5 to 8), with it being unreported in 5 patients (41.7%).

### Primary Smile Salvation Procedures and House-Brackmann Score

A total of 7 different techniques were utilized across the review for primary smile salvation; gracilis muscle free flap (GMFF)+cross-facial nerve graft (CFNG) (n=4), GMFF+masseteric nerve coaptation (MNC) (n=3), with an additional patient receiving a static midface fascia lata sling (SMFLS) alongside the GMFF+MNC (n=1), GMFF+ipsilateral intrapetrous facial nerve graft (IIFNG) (n=1), transverse rectus abdominis myocutaneous (TRAM) flap+facial nerve cable graft (FNCG) connected by a sural nerve graft (n=1), temporalis pedicled flap (TPF) (n=1), and latissimus dorsi free flap (LDFF)+CFNG (n=1). The House-Brackmann score following primary salvation procedures was VI in 11 patients (91.7%) and IV in one patient (8.3%), resulting in a mean score of 5.83.

### Secondary Smile Salvation Procedures and House-Brackmann Score

A total of 8 secondary smile salvation techniques following a failed previous attempt were identified. Patients 8, 9, and 10 (25%) underwent revision of a GMFF that had initially been innervated by a CFNG. The flap vasculature (facial artery and vein) was preserved, and the primary flap was reinnervated through end-to-end anastomosis with the masseteric nerve.^24^ Patients 2 and 11 (16.6%) received a second GMFF, harvested from the contralateral leg; the flap was revascularised to the same recipient vessels used in the first procedure, and reinnervated by the masseteric nerve through end-to-end anastomosis. Importantly, the masseteric nerve had not been used in the initial attempt.^16,22^ Patients 5 and 6 (16.6%) underwent the same procedure with a second GMFF reinnervated by the masseteric nerve, although the masseteric nerve had also been utilized for innervation of the first GMFF. Patient 7 (8.3%) underwent the same revision, but the second GMFF was revascularised using contralateral cervical vessels, the exact names of which were undisclosed in the report. This case also included placement of a static midface fascia lata sling in combination with masseteric nerve coaptation.^23^ Concerning patient 1 (8.3%), an LDFF was revascularised to the artery and vein of a previous TRAM flap anastomosis and reinnervated by the posterior branch of the deep temporal nerve through end-to-end coaptation. A GMFF revascularised to the contralateral facial artery and vein, with masseteric end-to-end reinnervation, was used following failure of a temporalis pedicled flap in patient 3 (8.3%).^22^ Patient 4 (8.3%) underwent external neurolysis of a GMFF innervated by the masseteric nerve.^23^ Finally, patient 12 (8.3%) underwent revision using a GMFF that was revascularised to the transverse facial artery and vein, and reinnervated through the masseteric nerve with end-to-end anastomosis, following failure of an LDFF with CFNG.^16^

The House-Brackmann score following secondary salvation was III in 5 patients (patients 1, 3, 5, 11, and 12) (41.6%), II in 4 patients (patients 4, 6, 7, and 8) (33.3%), I in one patient (patient 2) (8.3%), and unreported in 2 patients (patients 8 and 9) (16.6%). The average House-Brackmann scores postsecondary salvation procedure was 2.4.

### Complications

Complications were poorly reported across the review and were unattainable in 7 patients (patients 1 to 7) (58.3%). Of the papers that reported adequate data, 4 patients (patients 9 to 12) (33.3%) experienced no complications, and 1 patient (patient 8) (8.3%) suffered a hematoma requiring surgical evacuation.

### Additional Procedures

Three studies reported additional procedures undergone by patients at a later date to the secondary salvation.^16,23,24^ Patients 4, 5, 6, and 10 (33.3%) received a static midface fascia lata sling, with patient 10 also undergoing flap thinning. In addition, patient 8 (8.3%) underwent flap thinning and canthoplasty. Patients 9, 11, and 12 (25%) received no additional procedures within the follow-up period of their respective studies. It was not reported if patients 1, 2, 3, and 7 (33%) underwent any additional procedures.

### Time to Reinnervation

The mean time until reinnervation of the secondary salvation procedure was 72.8 days (range 8 to 121.7) based on data from patients 4 to 12. The time taken until reinnervation was not reported in patients 1 to 3 (25%).

### Ability to Smile and Smile Type

All included patients were reported to have improvement in smile-related outcomes following secondary salvage; however, the degree to which this represented true dynamic smile restoration was inconsistently reported. A voluntary smile alone was documented in patients 6, 8, 9, 11, and 12 (41.6%), while patient 10 (8.3%) achieved both voluntary and spontaneous smiling. In the remaining 6 patients [patients 1, 2, 3, 4, 5 and 7 (50%)], the nature of the restored smile was not reported.

## DISCUSSION

This systematic review aimed to explore what is available in the current medical literature on the methods of secondary salvation of the smile, following a failed primary reanimation procedure for facial nerve paralysis. A number of key observations can be made following the review; however, these findings are undermined by several critical limitations.

The review emphasizes the absence of a gold-standard technique for secondary smile reanimation in facial nerve paralysis, as evidenced by the identification of 8 distinct techniques across only 12 patients. Despite the heterogeneity of approaches, all patients demonstrated improvement in smile-related outcomes following secondary procedures. However, this should be interpreted cautiously, as the included studies did not consistently distinguish between dynamic smile restoration and static improvement in facial symmetry. Only one patient was reported to regain both voluntary and spontaneous smiling, while voluntary smiling alone was reported in 5 patients. In several cases, static adjunctive procedures such as fascia lata slings were used, which may improve oral commissure position and smile symmetry without necessarily restoring dynamic, emotionally driven smiling.

Except for 2 cases (patients 9 and 10, in whom postprocedure House-Brackmann scores were unattainable), all patients demonstrated improvement in House-Brackmann score, compared with their outcomes after the primary intervention. These findings suggest that smile restoration, and facial reanimation remain feasible, even after unsuccessful primary attempts involving complex procedures such as free flaps. Another notable observation from this review is that the GMFF was the most commonly used secondary technique following failed free flap procedures for smile restoration. Of 8 patients receiving a second free flap, 7 had a GMFF. Interestingly, a GMFF was used regardless of whether the primary reconstruction had also been a GMFF or an alternative flap, such as the LDFF or TPF. This finding highlights the reliability of the GMFF in restoring smile function in patients with facial nerve palsy, both after initial GMFF failure, and following the unsuccessful use of other flaps. All patients achieving the ability to smile after the secondary reanimation suggests that the GMFF may represent the optimal free flap option for salvage procedures in smile reanimation; however, due to the limitations of this review, no conclusive statements can be made.

A further key observation is that the masseteric nerve was used for reinnervation in all but one case (83.3%), and importantly, it was utilized in multiple contexts. Eisenhardt et al^24^ described secondary reinnervation of a prior GMFF, using the masseteric nerve after failure of a CFNG in 3 patients, suggesting that successful smile restoration may be achieved by altering the innervation of the primary flap, rather than resorting to an additional free flap. Conversely, in cases where an additional free flap was required, the masseteric nerve was also used for reinnervation, even when it had failed to provide adequate function in the primary flap, as reported by Mohanty et al^23^ in patients 5 to 7. Finally, Ibarra et al^22^ and Bianchi et al^16^ demonstrated that the masseteric nerve could achieve successful reinnervation in secondary free flap procedures, when the primary flap had failed with innervation by alternative donor nerves (patients 2, 11, and 12). Collectively, these findings highlight the versatility of the masseteric nerve in secondary reanimation procedures and suggest that it may represent the most reliable option for reinnervation when secondary salvation is required.

An additional encouraging finding of the review is the successful use of the primary procedure’s artery and vein anastomosis in the secondary salvage surgery. This was observed in patients 1, 2, 5, and 6, all of whom had the anastomosis from their previous failed GMFF utilized for their secondary procedure, with the exception of patient 1, whose primary reconstruction had been a TRAM flap. This is particularly encouraging as it demonstrates the technical feasibility and reliability of reusing recipient vessels, even after a failed free flap. The ability to employ the same vascular pedicle reduces the need to identify alternative recipient sites, and preserves further reconstructive options, should additional salvage procedures be required. Finally, it is also noteworthy that external neurolysis was performed in patient 4, who demonstrated the shortest time to reinnervation. This suggests that, when successful, neurolysis may provide the most rapid route to smile restoration following a failed primary procedure.

The authors were unable to identify a systematic review employing a comparable methodology. However, Kollar and colleagues conducted a retrospective cohort study addressing a similar topic. Their cohort comprised 12 patients who had previously undergone static procedures (n=6), temporalis transfer (n=2), or GMFF (n=4) as the primary intervention. Consistent with the present review, Kollar and colleagues reported improvements across all static and dynamic midface parameters following secondary GMFF with masseteric nerve reinnervation (n=9). Notably, their study focused exclusively on GMFF as a secondary salvage procedure, and did not evaluate alternative strategies, such as secondary reinnervation of a primary flap with the masseteric nerve. This underscores the need for an up-to-date comprehensive review encompassing all available techniques currently described in the literature.^18^

Several critical limitations undermine the findings of this review. Due to inconsistencies across the review in outcome reporting, external expertise was sought for the interpretation of certain data. Postoperative outcomes for patients 4, 5, and 7 were reported as philtral deviation from the midline at rest, and the degree of philtral deviation correction from baseline (mm); these values were approximated to the corresponding House-Brackmann score. These inconsistencies undermine the generalizability and accuracy of the review.

In addition, the scarcity of eligible studies resulted in a small sample size of only 12 patients, substantially limiting the reliability, accuracy, and generalizability of the observations, as conclusions drawn from such a small cohort are unlikely to represent the broader population. Internal validity was further restricted by the absence of randomized controlled trials (RCTs) on secondary smile salvation, necessitating the inclusion of lower-level evidence such as case series, which are highly susceptible to bias. In addition, Ibarra and colleagues and Mohanty and colleagues did not report the duration of follow-up, while Eisenhardt and colleagues and Bianchi and colleagues reported relatively short mean follow-up periods of 6 and 10 months, respectively. This limited the review’s ability to assess long-term efficacy and increases the risk of the observations made in this review being inaccurate. External validity was also impaired through the incomplete reporting of key clinical outcomes, including complications, additional procedures, time to reinnervation, and the type of smile restored. As a result, the review was limited to describing existing techniques rather than identifying an optimal approach. These limitations highlight the need for future high-quality studies, particularly long-term RCTs in patients with facial nerve palsy undergoing secondary smile restoration after failed primary procedures, with comprehensive reporting of study design, patient characteristics, and clinical outcomes.

## CONCLUSION

In conclusion, this review identified a number of techniques in the current literature that may be used to restore the smile following a failed primary procedure. Some approaches appear particularly promising, including the frequent use of gracilis muscle free flaps and the diverse applications of the masseteric nerve. Encouraging outcomes were noted, including reported improvement in House-Brackmann score and smile-related function or symmetry across all included patients. However, true dynamic smile restoration was incompletely reported, with only one patient clearly achieving both voluntary and spontaneous smiling. Moreover, these findings are further limited by the paucity of available evidence, and methodological shortcomings of the existing studies, particularly the incomplete reporting of data and inconsistencies in outcome measures. Future research should prioritize long-term randomized controlled trials investigating secondary smile reanimation, with rigorous reporting of study design, patient characteristics, and comprehensive clinical outcomes, including complications, reinnervation time, and smile quality.
